# Gradients of *Eph-A6* expression in primate retina suggest roles in both vascular and axon guidance

**Published:** 2009-12-09

**Authors:** Peter Kozulin, Riccardo Natoli, Michele C. Madigan, Keely M. Bumsted O’Brien, Jan M. Provis

**Affiliations:** 1 ARC Centre of Excellence in Vision Science and Research School of Biology, The Australian National University, Canberra, Australia; 2 School of Optometry and Vision Science, The University of New South Wales, Kensington, Australia; 3 Save Sight Institute, The University of Sydney, Sydney, Australia; 4 Australian National University Medical School, The Australian National University, Canberra, Australia

## Abstract

**Purpose:**

Recently we identified high levels of expression of *Eph-A6* in the macula of developing human retina and showed localization of *Eph-A6* to ganglion cells (GC). In the present study we investigated the expression of some members of the ephrin family in developing primate retina, including the topography of *Eph-A6* expression, and its ligands, in developing macaque retinas.

**Methods:**

We extracted RNA from human fetal retinas and probed for *Eph-A5–A7*, *Eph-B1*, *ephrin-B2,* and *ephrin-A1-A5* by RT–PCR, then prepared riboprobes for *Eph-A5-A7*, *Eph-B1* and *ephrin-A1, -A4* and *-B2*. Paraffin sections of fetal macaque retinas were used to localize expression of Ephs and ephrins by in situ hybridization and immunohistochemistry.

**Results:**

We identified prominent gradients of *Eph-A6* mRNA expression in the ganglion cell layer (GCL) of fetal macaque retinas of different ages. The gradient of *Eph-A6* expression was high near the optic disc and low at the developing macula at fetal day (Fd) 55. At Fd 70 and 80, the gradient of *Eph-A6* expression was reversed, being higher temporal to the macula, and low at the disc. By Fd 110, when the fovea begins to form, a pattern of expression was established that persisted into the postnatal period, in which the highest levels of expression were detected at the developing fovea, and progressively lower levels of expression were detected at increasing distance from the fovea. Beginning at Fd 70, we also detected a gradient of *Eph-A6* expression running perpendicular to the retinal surface within the GCL of central retina that was high in the inner GCL and low in the outer GCL. This second pattern persisted into the neonatal period. We found the two ligands for *Eph-A6*, *ephrin-A1* and *ephrin-A4*, expressed by *Pax2*-immunoreactive astrocytes, in the optic nerve head and in the retina, by in situ hybridization and immunohistochemistry. We propose that during development of the retinal vasculature, migration of ligand-bearing astrocytes is slowed along this *Eph-A6* expression gradient through repellent *Eph-A6* - *ephrin-A1* and *-A4* signaling.

**Conclusions:**

Patterns of *Eph-A6* expression in the developing macaque retina suggest that *Eph-A6 - ephrin-A1* and *-A4* repellent signaling has a role in retinal vascular patterning, and in the postnatal maintenance of projections from macular and foveal GC.

## Introduction

The fovea centralis (fovea) is formed in temporal retina during late fetal life by the centrifugal displacement of cells of the inner retina, leaving cone cell bodies lining the floor of the pit. The fovea is centrally placed in the less well defined “macula.” The macula is a region approximately 3 mm in diameter recognized clinically by its high concentration of yellow, xanthophyll pigments (hence, macula lutea) and a relative absence of large diameter retinal vessels. In this region, the neural retina is also highly specialized: the density of all neural elements is elevated; there is a peak spatial density of cone photoreceptors; there are no rods; and there is a prevalence of “midget” or “parvocellular’ circuitry [1-3]. Signals encoded by these circuits provide the brain with the detailed spatial and color information, characteristic of macular visual function. Absence of large retinal vessels from the macula is an adaptive advantage, since large vessels cause “shadowing” of the cone mosaic [4] and if present, would degrade the quality of the image that strikes the high density cone array.

Under normal circumstances the foveal region is never vascularized [5] and remains so throughout life. Despite this, the principal retinal angiogenic factor, vascular endothelial growth factor (VEGF), is highly expressed at the macula during development of the retinal vessels [6,7], so it is a paradox that macular vessels are slow to develop [8,9]. These observations led us to the hypothesis that repellent guidance cues might inhibit the migration of endothelial cells, and astrocytes, into the macular region during development.

Three lines of evidence led us to investigate the expression patterns of Eph receptors and their ligands. First, Eph receptor signaling has an important role in retinal axon pathfinding [10-13] and retinal vessels follow trajectories similar to those taken by retinal axons [14,15]. Second, development of retinal vessels is dependent on Eph receptor signaling [16-19]. Third, in a microarray analysis of differential gene expression in the developing human macula [20], we found higher levels of expression of *Eph-A6* in the macula, confirmed by quantitative RT–PCR. In the present study we aimed to identify the expression patterns of *Eph-A6* and its ligands in the developing macaque retina.

## Methods

### Specimens

We used histological sections of retinas from 10 macaques at fetal days (Fd) 55, 64, 70, 80, 110, 115, 120, 145, postnatal day (P) 5, and P 3 months. This developmental range straddles the period during which the perifoveal capillaries form and the foveal avascular area is defined, that is, between about Fd 90 and several days postnatal [9]. The sections were obtained by A.E. Hendrickson from the breeding colony of the Primate Center at Bogor Agricultural University, (Bogor, Indonesia), using protocols approved by the University of Washington (Seattle) Animal Care Committee and in compliance with guidelines of the Association for Research in Vision and Ophthalmology. Fetuses were delivered by aseptic caesarean section, euthanized by intraperitoneal overdose of barbiturate, and then enucleated. Eyes were immediately injected with methyl Carnoy’s fixative, which contains a 6:3:1 ratio of methyl alcohol: acetic acid: chloroform. Specimens were then fixed whole by immersion for 1–2 h, as described previously [21]. Sections were embedded in paraffin, and then 8 μm slices were cut. Only sections passing through the optic disc and foveal region were used for quantitative analysis in this study.

For RNA, four human retinas aged between 17 and 20 weeks of gestation (WG) were obtained at surgery to terminate pregnancy, with informed maternal consent. Ethical approval was obtained from the Human Ethics Committees of the University of Sydney and The Australian National University. Gestational age was determined by ultrasound before surgery and confirmed by postmortem measurements of foot length. RNA was extracted from donor retinas 90–120 min postmortem. A fifth human retina of 13 WG, the period immediately preceding appearance of vessels at the optic disc, was also obtained under similar circumstances for immunohistochemical analysis. This eye was fixed in 4% paraformaldehyde, embedded in paraffin, and processed in the same way as macaque eyes.

### PCR and in situ hybridization

Consistent with a previous study [21], we used RNA extracted from fetal human retinas for RT–PCR, because it was not possible to obtain fresh macaque retinas for RNA extraction. Whole retinas were excised, homogenized, combined with Trizol Reagent (Invitrogen, Carlsbad, CA) and chloroform, then purified and isolated using RNAqueous-Micro RNA isolation kit (Ambion, Inc., Austin, TX). In some cases, we used a 5 mm trephine to obtain a biopsy of the developing macular region, and extracted RNA from the macular sample as well as from the remaining retina (“surround” sample). First-strand cDNA synthesis was performed in a 20 μl reaction mixture using 1 μg RNA, 500 ng oligo (dT)_18_ primer, and 200 U SuperScript III reverse transcriptase (Invitrogen). CDNA was probed for expression of *Eph-A5*, *-A6*, *-A7*, *-B1*, and *ephrins -A1*, *-A2*, *-A3*, *-A4*, *-A5*, and *-B2*, using primers shown in Table 1. PCR products were visualized with electrophoresis using ethidium bromide staining on a 1% agarose gel.

**Table 1:** PCR Primers.

**Entrez Gene ID**	**Gene**	**Primers (5′-3′)**
2044	*Eph-A5*	F: ACCGATGAACCTCCCAAAAT
		R: TAGCCTGCTGCTGTACGTG
285220	*Eph-A6*	F: TGGGATGCCATCACTGAAAT
		R: CAAGAGGAACCAGCCAATCT
2045	*Eph-A7*	F: GATCCCAGAGGCTCTTTGC
		R: TTGATGGACAGCTCTATTTGG
2047	*Eph-B1*	F: AGCTGCTCCCGCTGTGAC
		R: TGCTCCGATGACCTCTTCA
1942	*ephrin-A1*	F: TCCGGAATGAGGACTACACC
		R: AGTGCCTGTCCCTCTCTTCA
1943	*ephrin-A2*	F: GCTGCTGCTCCTGCTGTTAC
		R: CTCGCCGTTGACCATGTA
1944	*ephrin-A3*	F: CCTGCACTGGAAGTGTCTGA
		R: ACCAGGAGTCAGGGAAAGGT
1945	*ephrin-A4*	F: TGGAGAGAGTGGCACATCAG
		R: GGAGGGCAACAAAAAGATGA
1946	*ephrin-A5*	F: GATGTGTGTGTTCAGCCAGG
		R: AGGGAGGCAGGAACAAGTTT
1948	*ephrin-B2*	F: TAACCAGGAGGGAGGGGTGT
		R: GCCGAATGCTACAAGACTAGG

PCR of several A-class Ephs and ephrins as well as *Eph-B1* and *ephrin-B2* was run using cDNA from four fetal human retinas of 17–20 WG. The PCR products for *Eph-A5*, *-A6*, *-A7*, *-B1* and *ephrin-A1*, *-A4,* and *-B2* were then used to generate DIG-labeled riboprobes for in situ hybridization.

We cloned and generated digoxigenin (DIG)-labeled riboprobes from PCR products for *Eph-A5*, *-A6*, *-B1*, *-A7* and *ephrin-A1*, *-A4,* and *-B2*. Briefly, cDNA fragments were amplified from total RNA of human fetal retina, and cloned using the pGEM-T DNA vector system and JM109 competent cells (#A3600; Promega, Madison, WI). The plasmid constructs containing the DNA fragments were purified, sequenced, and linearized using Nco I, Not I, and Sac II restriction enzymes (Promega). A DIG RNA Labeling Kit SP6/T7 (#1175025; Roche, Basel, Switzerland), which contains DIG-11-UTP, was used to transcribe the linearized plasmid and produce the antisense and sense probes.

Paraffin sections of macaque retina were placed in xylene (#XA003; Chem-Supply, Gillman, Australia) for 10 min for dewaxing. They were rehydrated in graded ethanols (2×100%, 90%, 70%, and 50%) and rinsed in 0.9% NaCl then phosphate buffered saline (PBS), which contained 0.8% NaCl in phosphate buffer, pH 7.4. The sections were fixed in 10% neutral buffered formalin (NBF; #90245; Merck, Darmstadt, Germany) for 20 min, washed in PBS (2×5 min), then immersed in 50 mM Tris–HCl, 5 mM EDTA (TE; pH 8.0) containing 20 μg/ml of Proteinase K (#3115828; Roche) for 7 min at 37 °C. Following a rinse in PBS and re-fixation in NBF for 20 min, the sections were immersed in a solution containing 0.1 M triethanolamine (#T9534; pH 8.0; Sigma, St. Louis, MO) and 2.5% acetic anhydride (#A6404; Sigma) for 10 min. After a rinse in PBS then 0.9% NaCl, the slides were dehydrated in graded ethanols and allowed to air dry.

Prior to hybridization, coverslipped sections were incubated for at least 1 h at 55 °C in prehybridization solution that comprised the following ingredients: 50% deionized formamide, 20% dextran sulfate (#0198; Amresco, Solon, OH), 500 µg/ml polyadenylic acid potassium salt (#P9403; Sigma), 50 µg/ml yeast t-RNA (#R8759; Sigma), 50 mM dithiothreitol (#D9779; Sigma); and a salt solution that contained 300 mM NaCl, 10 mM Tris base (#T6066; Sigma), 10 mM sodium phosphate (Na_2_HPO_4_; #S3264; Sigma), 5 mM NaEDTA, 0.2% Ficoll 400 (#F2637; Sigma), and 0.2% polyvinylpyrrolidone (#P5288; Sigma). For hybridization, each riboprobe was resuspended in hybridization solution at about 250 ng/ml. The coverslip and prehybridization solution was carefully removed after this incubation and the hybridization solution (prehybridization buffer containing 250 ng/ml riboprobe), heated to 55 °C, was applied to the sections with a new coverslip. The riboprobes were allowed to hybridize to the tissue overnight at 55 °C after which the coverslips were removed and the sections rinsed in 4× saline sodium citrate (SSC, pH 7.4), then washed in 2× SSC, 1× SSC and two washes of 0.1× SSC for 2 h at the optimal post-hybridization wash temperature (60–65 °C). A final wash of 0.1× SSC at room temperature for 10 min was also applied. The optimal post-hybridization wash temperature was that which produced the least amount of background labeling. For the *Eph-A6* probes, the optimal hybridization temperature was 55 °C, with post-hybridization washes done at 65 °C. For *ephrin-A1* and *–A4*, optimal hybridization and wash temperatures were 55 °C and 62 °C, respectively.

The slides were rinsed in washing buffer, which contained 0.10 M maleic acid, 0.15 M NaCl, and 0.3% Tween-20 (#0777; pH 7.5; Amresco). From there, they were immersed in blocking reagent solution (#1096176; 10% w/v; Roche) for 30 min. The sections were removed from the blocking solution and an anti-DIG-alkaline phosphatase antibody (#1093274 Roche) diluted to 1:1,000 was applied to the tissue and allowed to incubate for 30 min. The slides were subsequently rinsed in washing buffer (3×40 min) then placed for 5 min in a detection buffer that consisted of 100 mM Tris-HCl, 100 mM NaCl, 10 mM MgCl_2_, pH 8.0. Following hybridization to detect *Eph-A6* expression, riboprobes visualized by incubation for 5 min at room temperature in an HNPP fluorescent detection set (#1758888; Roche) using a Fast Red TR fluorescent substrate with a peak emission at 584 nm. The color reaction was stopped by washing in deionized water for 10 min. Riboprobes detecting ephrin-A expression were visualized by incubation in NBT/BCIP color substrate (#1681451; Roche) for 16 h at 4 °C. A similar detection buffer as that used before HNPP/Fast Red TR fluorescent color detection was used except the MgCl_2_ was omitted. The color reaction was stopped by washing in deionized water for 10 min, then preserved by fixing in NBF for 4 h at 4 °C. All sections were mounted in glycerol, coverslipped, and sealed with fingernail polish. The sections were scanned immediately or stored at −20 °C and scanned within 24 h (Fast Red) or 48 h (NBT/BCIP) after completion of the color reaction.

### Immunolabeling

Double immunolabeling using antibodies to *ephrin-A1* and *-A4* with anti-paired box-2 (*Pax2)* was used to localize ephrin proteins in the retina. Nonimmune goat IgG was used as a control for ephrin immunoreactivity. A list of antibodies used and their dilutions is shown in Table 2. Briefly, sections were dewaxed in xylene for 10 min and rehydrated in graded ethanols (2×100%, 90%, 70%, and 50%), then rinsed in deionized water followed by 0.3% Revealit-Ag (#AR1002; ImmunoSolution, Newcastle, Australia) in PBS (PBSR). The sections were then incubated in Revealit-Ag for 1 h at 37 °C for antigen retrieval and subsequently washed in PBSR for 5 min. Sections were blocked in 10% serum (diluted in PBSR) for 1 h at 37 °C, and incubated in primary antibody overnight at 4 °C. To avoid cross-reactivity, we applied primary antibodies raised in different species to the tissue consecutively. After thorough rinsing, ephrin (and control goat IgG) binding was visualized following 1.5 h incubation in a biotinylated secondary antibody then 1.5 h incubation in streptavidin Alexa 488. *Pax2* binding was visualized using Alexa 594 conjugated to an anti-rabbit secondary antibody (4 h incubation). Secondary antibodies and labels were allowed to incubate at 37 °C, and all antibodies were diluted in PBSR. Sections were rinsed in PBSR for 2×5 min after each incubation to remove unbound antibodies.

**Table 2:** Antibodies and immunoreagents.

**Antibody**	**Dilution**	**Manufacturer**
**Primary antibodies**		
Anti-mouse ephrin-A1 (goat, poly)	1:2,500**	R&D Systems
Anti-human ephrin-A4 (goat, poly)	1:2,500**	R&D Systems
Anti Paired Box-2 (Pax2; rabbit, poly)*	1:100	Covance
Control antibody		
Normal goat IgG	1:25,000**	R&D Systems
**Secondary antibodies**		
Biotinylated horse anti-goat (0.3% PBSR)	1:200	Zymed
Biotinylated goat anti-rabbit (0.3% PBSR)	1:200	Zymed
**Visualization**		
Conjugated goat anti-rabbit Alexa 594 (0.3% PBSR)	1:1,000	Invitrogen
Streptavidin Alexa 488	1:1,000	Invitrogen

Antibodies raised against the two ligands to *Eph-A6*, *ephrin-A1,* and *-A4*, were used to localize these proteins in sections of fetal macaque retina. Anti-*Pax2* double immunolabel was used to localize immature astrocytes, while background labeling was visualized using normal goat IgG antibody. *Pax2 is cross-reactive to human and other species. **IgG concentration applied to tissue is equal to ephrin antibodies given the reconstituted antibodies stocks were 0.1 mg/ml for ephrins and 1 mg/ml for IgG.

In some cases immunohistochemistry to detect *Pax2* expression was performed post-hybridization for *ephrin-A1* and *-A4*. After a PBS wash (2×5 min), the ephrin-A in situ hybridization (ISH) sections were blocked in 10% normal goat serum for 30 min and incubated with anti-*Pax2* double label overnight at 4 °C. *Pax2* labeling was visualized with 1 h incubation in a biotinylated secondary antibody followed by 30 min incubation in streptavidin Alexa 488. The serum and antibodies were diluted in PBS, and the blocking and secondary antibodies were allowed to incubate with the tissue at room temperature. The sections were washed in PBS for 5 min after each incubation period. All sections subjected to immunohistochemistry were mounted as per the in situ hybridization protocol.

### Imaging and optical densitometry

Sections were imaged using a Zeiss LSM 5 Pascal confocal microscope system and Pascal version 4.0 software (Jena, Germany). The microscope was equipped with LASOS HeNe 543 nm and Argon 488 nm scanning lasers (Jena, Germany). Hybridized sections were viewed within 24 h to avoid errors due to spread of the color reaction product.

### Image collection

For montages, we used a 20× objective and scanned a field size of 450×340 µm using standardized laser settings and optical sectioning. The full resolution images were exported, converted to grayscale for optical densitometry, and assembled as a montage using Adobe Photoshop (version 10.0.1). For immunolabeling, different objective lenses (20× or 40×), scan field sizes and optical section stack sizes were used according to the distribution of labeling. For optical densitometry we used a 40× objective and a frame size of 1024×1024 pixels, sampling a field 210×210 μm, and optical sectioning at a constant interval of 0.4 µm to create an image stack of 3.5 µm thickness. Z-sections were merged using the “projection” feature on the Pascal software. Settings for laser transmission, scan speed and direction, signal averaging, pinhole size, and optimal detector and amplifier gains were held constant during collection of all images from a single labeled section so that valid comparisons of labeling intensity could be performed. For printed viewing, the immuno images and images used for optical densitometry analysis were compiled in Photoshop in CMYK format and adjusted for optimal brightness and contrast.

### Optical densitometry

Quantitative analyses were performed on five sample regions temporal to the optic disc [21]: immediately adjacent to the optic disc (OD); nasal to the macula (nasal), at the mid-point between the OD and developing fovea; on the nasal foveal rim (rim); in the center of the foveal cone mosaic (fovea); and temporal to the fovea (temporal), such that the nasal and temporal samples were approximately equidistant from the fovea. Optical densitometry was performed using ImageJ (version 1.38x; National Institutes of Health, Rockville, MD) on representative sections from one specimen at each developmental age.

### Profiles of *Eph-A6* expression

Images from the five locations in each retina were converted to grayscale, and optical density profiles of DIG-labeled mRNA were extracted using the rectangular selection tool (in ImageJ). To construct profiles showing *Eph-A6* expression across the full thickness of the retina, we inverted images so that the most intense labeling appears black. Where the fovea had not formed (younger than 115 days) the profiles show the average pixel intensity in each line of pixels parallel to the retinal surface. For older specimens with a fovea, the profile was constructed from a narrow sample area centered on the base of the pit. To control for background labeling of the antisense riboprobe, we calculated the final intensity data (displayed on the profile) as a ratio against the intensity of the labeling in the ganglion cell layer (GCL) of the control (sense probe) sections of the same specimen. The Mann–Whitney test was used to compare labeling intensity in inner and outer halves of the GCL, at fovea and rim locations.

### Labeling intensity in the GCL at different locations in a single specimen

We used optical densitometry to quantify the intensity of mRNA labeling in the GCL at different locations in each specimen, to characterize the observable expression gradients. In all samples, cell nuclei were not labeled (0 intensity). The range of pixel intensities in the cytoplasm (where mRNA is expressed) ranged from very low levels up to saturation. A histogram displaying the range and frequency of pixel intensities throughout each sample location was obtained using ImageJ, and the mode intensity was identified. Because we aimed to obtain objective measures of *brightness* in the mRNA labeling intensity (a parameter of mRNA expression) to identify locations with the highest levels of *Eph-A6* expression, we discarded pixel values falling below the mode. We compiled images of the full thickness of the GCL at each of the five sample locations from successfully hybridized sections. We then used ImageJ to extract the mean intensity of the remaining pixels in each line of an image of the GCL (one data point per line of pixels), in each sample location. This approach did not alter the relative mean levels of brightness between samples, only the standard deviations of the means. The Mann–Whitney test was used to compare mean pixel intensity values in the foveal and nasal locations in each section analyzed.

## Results

### RT–PCR and in situ hybridization

**Figure 1:**
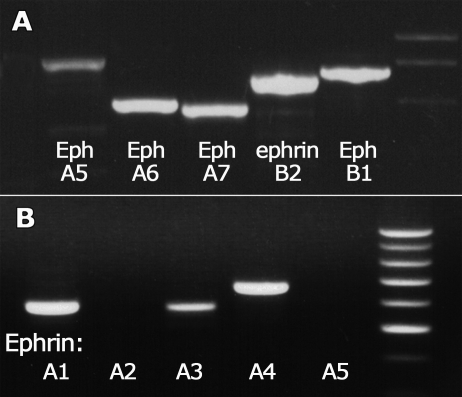
RT–PCR analysis of Eph and ephrin expression in 20 WG fetal human retina. **A**: We found low levels of amplification of *Eph-A5*, and (**B**) no amplification of *ephrin-A2* and *-A5*. Eph and ephrin PCR primers are listed in Table 1. PCR products were visualized with electrophoresis using ethidium bromide staining on a 1% agarose gel, and used to generate DIG labeled riboprobes for *Eph-A5, -A6, -B1, -A7*, and *ephrin-A1, -A4*, and *-B2*.

We detected expression of *Eph-A5*, *-A6*, *-A7* and *-B1*, and *ephrin-A1*, *-A3*, *-A4,* and *-B2* in cDNA from human fetal retinas (Figure 1). In situ hybridization showed expression of *Eph-A5* (data not shown) and *-A6* in the cellular layers of the retina, particularly the GCL. We did not obtain reliable results for *Eph-B1* or *ephrin-B2*. Although *Eph-A7* was detected by RT–PCR, *Eph-A7* mRNA was not visualized in the retina, similar to previous findings [22]. In contrast with a previous study [22], we did not detect *ephrin-A5* by RT–PCR. *Ephrin-A5* is expressed in peripheral retina and ciliary processes of the retina [22], which may have been deficient in the samples used for RT–PCR in the present study.

### *Eph-A6* expression

**Figure 2:**
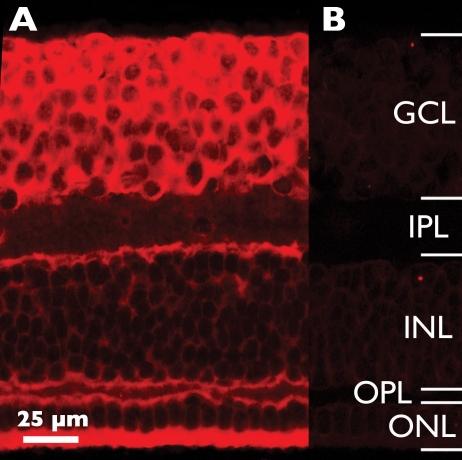
*Eph-A6* expression in Fd 110 macaque retina (foveal region) revealed by in situ hybridization. **A**: Antisense riboprobe strongly labeled the ganglion cell layer (GCL), particularly the innermost portion. **B**: Sense probe produced no specific labeling. Confocal gain and offset settings were the same in **A** and **B**. Abbreviations: inner nuclear layer (INL); inner plexiform layer (IPL); outer nuclear layer (ONL); outer plexiform layer (OPL).

**Figure 3:**
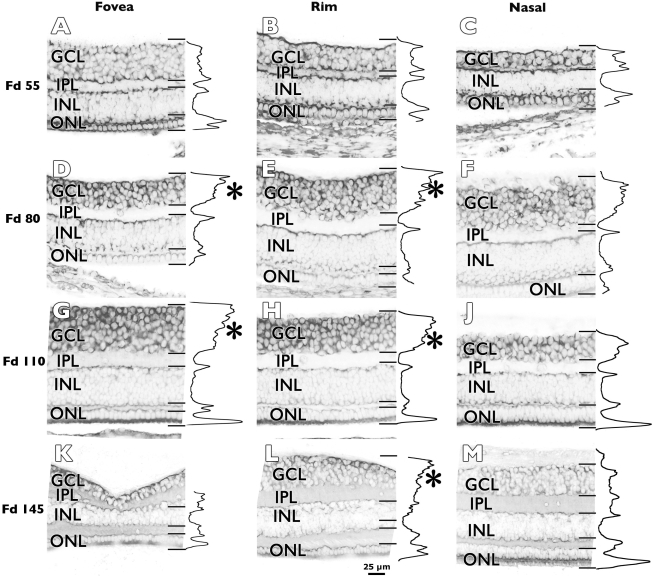
Optical densitometry profiles of *Eph-A6* mRNA expression. **A**-**C**: At Fd 55, *Eph-A6* is expressed in all cell layers at each location, but at higher levels in the outer nuclear layer (ONL) compared to other layers. **D**-**F**: By Fd 80, *Eph-A6* mRNA is higher in the ganglion cell layer (GCL) than other layers. The mRNA is also higher at the fovea and rim locations compared to nasal (**F**). At the fovea (**D**) and rim (**E**) locations there is a gradient of *Eph-A6* mRNA in the GCL, with significantly higher levels of mRNA labeling present in the inner GCL, and lower levels in the outer GCL (*p<0.0001, Mann–Whitney test). **G**-**J**: A similar gradient of mRNA expression was detected in the fovea and rim locations in a Fd 110 retina. **K**-**L**: *Eph-A6* mRNA expression remains relatively high in the GCL at Fd 145 at the fovea (**K**) and on the rim (**L**), where an inner-to-outer gradient of expression in the GCL is still detected. *Eph-A6* expression is relatively lower and uniform nasal to the fovea (**M**). Abbreviations: inner nuclear layer (INL); inner plexiform layer (IPL).

We detected *Eph-A6* mRNA expression in the cell layers of fetal macaque retinas (Figure 2). Unlike the other family members tested, the pattern of labeling for *Eph-A6* mRNA in the GCL varied as a function of both age and retinal location. This is illustrated in Figure 3 by comparing labeling patterns at three locations (fovea, rim, and nasal), at four different ages. At Fd 55 (Figure 3A-C) *Eph-A6* mRNA was found at low levels in the cellular layers, with the lowest levels detected in the middle of the inner nuclear layer, and the highest levels detected in the outer nuclear layer. By Fd 80 (Figure 3D-F) *Eph-A6* expression was much higher in the GCL than other layers, but low in the nasal location compared with fovea and rim, suggesting a gradient of expression across the retinal surface, centered on the fovea. By Fd 110, a strong, fovea-centered, gradient of *Eph-A6* expression was detected across the GCL such that *Eph-A6* expression was highest in the foveal GCL (Figure 3G), intermediate in the GCL on the foveal rim (Figure 3H) and lower in the nasal GCL (Figure 3J).

A second gradient of *Eph-A6* expression, running perpendicular to the retinal surface, was also detected in the GCL at fovea and rim locations at Fd 80 and Fd 110. At these ages *Eph-A6* expression is highest in the ganglion cells (GC) closest to the retinal surface, and considerably lower in GC adjacent to the inner plexiform layer. These gradients of *Eph-A6* expression were still distinct on the foveal rim at Fd 145 (Figure 3L), and are explored in greater detail as follows.

### Inner-to-outer GCL gradient of *Eph-A6* expression

Optical densitometry profiles of *Eph-A6* mRNA showed a gradient of *Eph-A6* mRNA expression across the depth of the GCL at Fd 80, 110, and 145 at fovea and rim locations (Figure 3 D-E,G-H,K-L), and are indicated in the densitometry profiles by asterisks (*). Highest levels of expression were detected in the inner GCL, and typically, the levels of *Eph-A6* mRNA in the outer GCL were approximately half that detected at the inner GCL margin. Labeling was generally low in the nerve fiber layer. This effect was also detected in the rim samples from specimens at P 5 days and P 3 months (data not shown).

**Figure 4:**
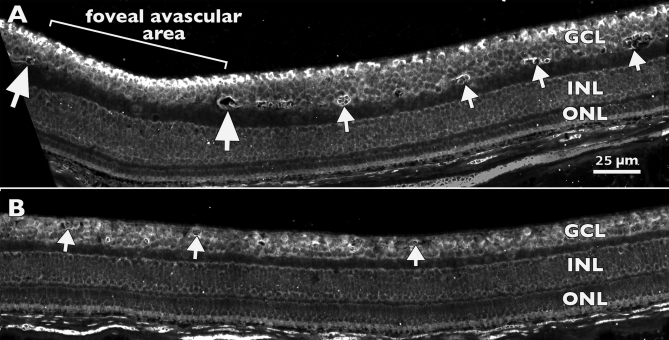
In situ hybridization for *Eph-A6* expression in a Fd 115 macaque retina. **A**: At the developing fovea and in adjacent temporal retina, peak *Eph-A6* mRNA expression is detected in the inner ganglion cell layer (GCL) within the foveal avascular area (bracket). Large arrows mark vessels at the inner margin of the perifoveal plexus. The area between the two large arrows is devoid of vessels. Smaller arrows indicate vessel profiles deep in the GCL (GCL plexus), which is a characteristic of macular vessels. **B**: Approximately 500 µm further into temporal retina, vessels (arrows) are present in the inner and outer GCL, and levels of *Eph-A6* mRNA in the GCL are lower, and more uniform across the depth of the GCL, compared with **A**. Together, the images show two gradients of *Eph-A6* expression: a high-to-low gradient from inner to outer GCL at the developing fovea (in **A**), and a high-to-low gradient from fovea to periphery (top left GCL in **A** to lower right GCL in **B**). Both **A** and **B** are at the same magnification. Abbreviations: inner nuclear layer (INL); outer nuclear layer (ONL).

In a specimen at Fd 115, when the perifoveal capillary plexus was becoming established around the foveal avascular zone, this gradient of *Eph-A6* expression was particularly prominent (Figure 4). In this retina there was a clear peak in *Eph-A6* mRNA expression in the inner GCL in the foveal avascular area, in which the fovea was beginning to form. On the margin of the foveal avascular area, which ultimately becomes the foveal rim, the gradient of high inner GCL and lower outer GCL expression of *Eph-A6* mRNA was also prominent. Capillaries that formed the perifoveal plexus were present deep in the GCL (arrows, Figure 4A). Less than a millimeter farther into temporal retina (Figure 4B) both the distribution of *Eph-A6* in the GCL and the placement of the blood vessels were quite different, such that *Eph-A6* levels in the GCL were somewhat lower than at the fovea and more uniform across the GCL. At this location the retinal vessels were also more superficially placed in the GCL (arrows, Figure 4B).

### *Eph-A6* expression varies with eccentricity

**Figure 5:**
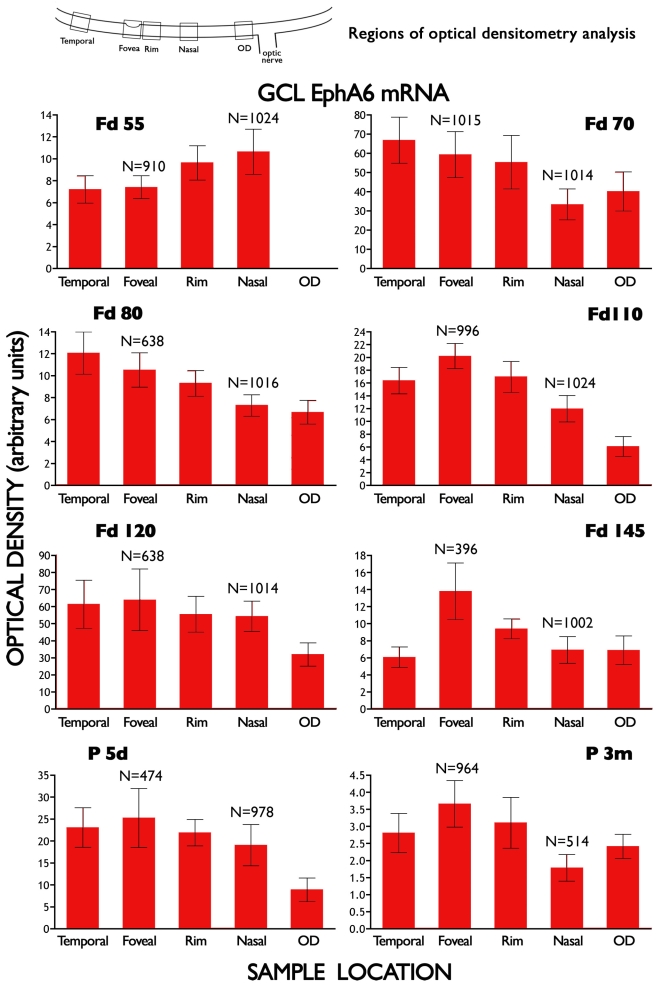
Mean optical densitometry measures of *Eph-A6* mRNA expression in the ganglion cell layer. Densitometry was performed at five sample locations (schematic) in eight retinas of different ages (one section quantified at each age). Note that the levels of expression (y-axis) are in arbitrary units, and not directly comparable between animals. The data show that the relative levels of *Eph-A6* at the five sample locations change between Fd 55 and Fd 80, but that by Fd 110 a pattern of expression is established where peak levels of *Eph-A6* mRNA are detected in the ganglion cell layer (GCL) at the fovea. There is a gradual decline in expression levels at locations increasingly distant from the fovea. Levels of mean *Eph-A6* optical densitometry measures were significantly different between foveal and nasal locations at all ages (Mann–Whitney, p<0.0001), where n=number of lines of pixels of the image analyzed (see Methods). Note that the Fd 55 section did not pass through the optic disc (OD).

The histograms in Figure 5 summarize the mean brightness of *Eph-A6* mRNA labeling in the GCL, at five sample locations, in eight retinas. The data showed a gradual change in the distribution of *Eph-A6* across the five locations between Fd 55 and P 3 m. At Fd 55, the highest levels of *Eph-A6* expression were detected between the fovea and optic disc (nasal), and the lowest levels were detected temporal to the fovea (temporal), with intermediate levels of expression at the fovea and on the rim. By Fd 70, the highest levels of *Eph-A6* were detected in the temporal sample, and the lowest levels near the OD. However, by Fd 110, when the fovea was forming and the foveal avascular area was being defined, a pattern of *Eph-A6* expression emerged that persisted into the postnatal period. That is, the highest levels of *Eph-A6* mRNA were detected in the GCL at the fovea, and there were lower levels of *Eph-A6* in the GCL temporal and nasal to the fovea (Figure 5; Fd 110, Fd 120, Fd 145, P 5 days and P 3 m). Thus, there was a gradient of *Eph-A6* expression in the GCL, in which peak expression occurred in the emerging fovea. The morphological appearance of this gradient of *Eph-A6* mRNA labeling is illustrated in Figure 4 (Fd 115), which is representative of the pattern of mRNA labeling seen in all specimens in this age range. As shown in Figure 4, by approximately 3 mm eccentricity (right hand side of Figure 4B), *Eph-A6* expression in the GCL was at very low levels, comparable with the remainder of the GCL. *Eph-A6* expression in the GCL was uniform and at low levels outside the analyzed region.

### *Ephrin-A1* and *-A4* localization

**Figure 6:**
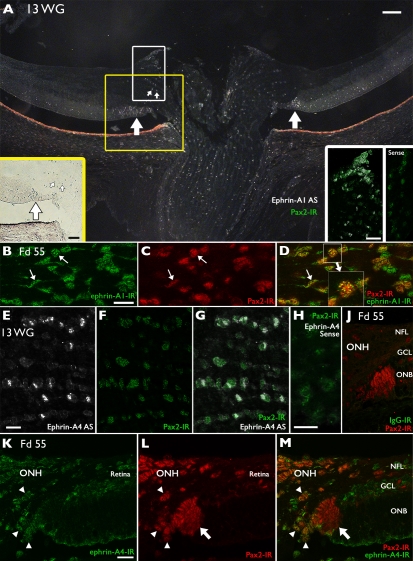
Localization of *ephrin-A1* (**A**-**D**) and *-A4* (**E**-**H**, **K**-**M**) in human and macaque retinas. **A**: Low magnification, dark field image of a section from a 13 WG human retina showing *ephrin-A1* mRNA expression (white) in cells distributed throughout the optic nerve head and immediately adjacent retina. The yellow box indicates the region shown in bright-field illumination (left inset). A triangular cluster of cells expressing *ephrin-A1* mRNA in the outer neuroblastic layer of the retina, adjacent to the optic nerve is denoted by a heavy arrow. These cells were also immunoreactive for *Pax2* (see **F** and **G**). *Ephrin-A1* expressing cells in the optic nerve head and adjacent to Bergmeister's papilla are indicated by the white box. The inset to the right shows colocalization of *ephrin-A1* (white) and *Pax2* (green) in these cells. The far right inset shows cells in the same regions of the adjacent sense-treated section. **B**-**D**: High magnification image of the optic nerve in a Fd 55 macaque shows *ephrin-A1* immunoreactivity (green) on the plasma membrane of cells (arrows) whose nuclei were *Pax2* immunoreactive (red), indicating the cells are astrocytes. Inset in **D** shows a high magnification view of an astrocyte with punctate-like *ephrin-A1* immunolabeling on its plasma membrane. **E**-**G**: Dark field scan of the optic nerve head showing *ephrin-A4* mRNA expression (white) in *Pax2*-positive astrocytic cells (green) in the 13 WG human retina. **H**: The *ephrin-A4* sense probe showed no specific labeling in a similar field in the optic nerve head. **J**: Goat IgG, control serum for *ephrin-A1* and *-A4* (Alexa 488), did not label astrocytes in Fd 55 tissue. **K**: At Fd 55, *ephrin-A4* immunoreactivity (green) was detected in the optic nerve head (ONH) and adjacent retina. **L**-**M**: Double labeling with anti-*Pax2* (red) shows *ephrin-A4* immunoreactivity surrounding the *Pax2* immunoreactive nuclei, indicating they are astrocytes (arrowheads). Astrocytes in the outer neuroblastic layer at the retina-optic nerve junction are *Pax2* immunoreactive (large arrow), and cells on the outer margin of this population are also *ephrin-A4* immunoreactive. Scale bars in **A** represent 50 µm. Scale bars in panels **B**-**M** represent 25 µm. Abbreviations: antisense (AS); ganglion cell layer (GCL); immunoreactivity (IR); nerve fiber layer (NFL); outer neuroblastic layer (ONB); optic nerve head (ONH).

Using RT–PCR, we detected three ephrin-A ligands in the retina: A1, A3 and A4 (Figure 1B). Cells expressing the ligands of *Eph-A6* - *ephrin-A1* and *ephrin-A4* [23] were identified in fetal primate retina using in situ hybridization and immunolabeling (Figure 6). Limited tissue resources, combined with difficulties localizing astrocytes in the GCL of hybridized sections due to their small size and sparse distribution, prevented a detailed analysis of these cells in the vicinity of the developing fovea. Rather we focused on the optic nerve head, where astrocytes are found at higher densities, and in a relatively cell-sparse environment, compared with the retina.

*Ephrin-A1* and *-A4* mRNA was identified in cells in the optic nerve head in a 13 WG human retina (Figure 6A,E,G). These cells were distributed throughout the proximal optic nerve and optic nerve head, showing some encroachment into the adjacent nerve fiber layer (NFL). Counter-immunolabeling with antibody against *Pax2* showed colocalization of mRNA and *Pax2* in these cells, indicative of immature or migrating astrocytes [24]. The dark field image (Figure 6A) shows the distribution of *ephrin-A1* mRNA-containing cells (small arrows) in the optic nerve head and adjacent retina. A triangular-shaped cluster of *ephrin-A1* expressing cells can also be seen adjacent to the optic nerve head, at the optic nerve-retinal boundary (Figure 6A, heavy arrows). The inset to the left shows this region in bright-field illumination. The inset to the right shows colocalization of *Pax2* (green) and *ephrin-A1* (white) on the margin of the optic nerve head and Bergmeister's papilla (white box). A similar pattern of expression was observed for *ephrin-A4*, which also colocalized with *Pax2* (Figure 6E-G). The sense probes showed no specific labeling (Figure 6A far right inset, Figure 6H).

We also detected moderate immunoreactivity for *ephrin-A1* and *-A4* in the optic nerve head and in the retinal ventricular zone adjacent to the optic nerve head, and in Bergmeister’s papilla in fetal macaque retinas at Fd 55 (Figure 6B-D, K-M), Fd 64, and Fd 70 (not shown). These *ephrin-A1* and *-A4-*immunoreactive cells were also immunoreactive for *Pax2*, although some *Pax2*-immunoreactive cells did not express these ephrins. Specimens incubated in nonimmune IgG of the same class as the ephrin-A antibodies showed no labeling (Figure 6J).

## Discussion

In humans, macaques, and marmosets, the retinal vasculature develops relatively late, starting at around 14 WG in humans, Fd 70 in macaques and Fd 100 in marmosets. On the nasal side of the optic disc, vessels grow in a radial fashion toward the periphery, similar to the pattern of growth seen in rodent models. However, temporal to the optic disc the developing vessels avoid the macular region, which they skirt around in their trajectory toward the periphery [25-31], and growth of vessels along the horizontal meridian, and in the vicinity of the fovea is very slow [8,9]. While the macula is developmentally the most advanced region of the neural retina, during the early phase of vascular outgrowth it remains avascular for a prolonged period, so that completion of the capillary plexus that surrounds the fovea is delayed into the neonatal period in humans and monkeys (reviewed in [7,31]). The factors responsible for the delayed development of the perifoveal capillaries have not been identified. The present results show gradients of mRNA expression for the axon guidance receptor *Eph-A6* in the GCL of macaque retina that coincide with key phases of retinal vascular development. We also find by in situ hybridization and immunohistochemistry the two ligands for *Eph-A6*, *ephrin-A1* and *-A4*, on *Pax2*-positive cells in the optic nerve head and nerve fiber layer consistent with the identification of astrocytes, which lead the developing vessels into the retina. These results implicate *Eph-A6* in vascular development in the primate macula.

We also detect high levels of *Eph-A6* expression in macular GC well into the postnatal period, after development of the retinal vasculature is completed. *Eph-A6* has been implicated in the prenatal guidance of axons to target nuclei during human development [22], and it has been suggested previously that these synaptic territories are “refined” postnatally [32]. We suggest that during postnatal refinement of synaptic territories, high levels of *Eph-A6* expression in macula may serve to “mark” GC that convey signals from the foveal region to the visual target nuclei.

### Eph receptor signaling

Eph tyrosine kinase receptors are divided into A and B classes according to the nature of the ephrin ligands they bind. Both classes of ligand-receptor pairs participate in chemoattractant and chemorepellent signaling, in either a forward direction–i.e., downstream signaling in the receptor-bearing cell–or in reverse, in which interactions with the receptor initiate changes upstream, in the ligand-bearing cell, directing cell movement, adhesion, and de-adhesion events. Repellent signaling along opposing gradients of ligands and receptors of both classes is the principal mechanism underpinning topographic mapping of the retina onto visual target nuclei, in a variety of species [10,33-39], including humans [22]. For comprehensive reviews, see [13,40,41].

Graded Eph-A expression has been described in many regions of the developing vertebrate central nervous system, including retina [22,35,36], superior colliculus [35,42,43], dorsal lateral geniculate nucleus [22,39,44,45], and cortex [46-48]. These and other studies show that accurate mapping of projections onto sensory centers depends on complementary gradients of ligands and receptors, particularly Ephs and ephrins, along with other factors including coordinated firing of cohorts of projecting neurons [39,49-51]. In addition, Eph receptors and ephrins are implicated in angiogenesis and vascular remodeling. Eph-B signaling has a role in arteriovenous differentiation in vitro, and vascular development in the periphery in vivo. B-class ligands and receptors have been identified in the central nervous system and retina [17,18], peripheral ganglia and nerves (reviewed in [52]). *Ephrin-B2*, expressed by arterial endothelial cells, interacts with *Eph-B4*, expressed by venous endothelial cells, resulting in bidirectional signaling that inhibits VEGF- and angiopoietin-1-induced endothelial cell proliferation and migration [52-55]. Different roles in vascular development have been identified for A-class ephrins and their receptors. *Ephrin-A1* is expressed at sites of vascular development during mouse embryogenesis [56] and acts as a chemoattractant for endothelial cells in vitro following an inflammatory signal [57].

### Significance of graded *Eph-A6* expression in primate retina

#### A role in vascular patterning?

The most prominent gradient of *Eph-A6* expression occurs as a function of eccentricity from the developing fovea, such that peak *Eph-A6* expression occurs at the fovea (0 eccentricity), with a gradual decline in expression with increasing eccentricity, out to about 3 mm. A second gradient runs perpendicular to the retinal surface and is confined to the GCL of the foveal region and immediately adjacent retina (to about 1.5 mm eccentricity). These gradients begin to emerge at about Fd 80, after retinal vessels have emerged from the optic disc, and during the period when there is slow growth of retinal vessels along the horizontal meridian [9] and in the macular region [8].

Prior to Fd 80 our data suggest that expression patterns of *Eph-A6* are quite different. At Fd 55, when the foveal region has just begun to differentiate, *Eph-A6* expression is higher near the optic disc than at the incipient fovea (Figure 5), consistent with a previous study of human retina at a comparable stage of development [22]. High expression of *Eph-A6* at the disc would inhibit migration of ligand-bearing astrocytes into the retina, resulting in their accumulation in the optic nerve head as well as in Bergmeister's papilla, which is invested in astrocytes [58] that express *ephrin-A1* and *-A4* (data not shown). At Fd 70 we find a relative drop in levels of *Eph-A6* expression at the optic disc (Figure 5). This corresponds with the earliest stages of retinal vessel formation in the macaque [28,31]; this relative decline in *Eph-A6* expression may be permissive to astrocytes entering the retina at this stage. However, because we have limited numbers of specimens in this early age range, these results require further clarification and confirmation.

Our most significant findings are from five animals, aged between Fd 110 and P5, during which period the foveal avascular region is defined, and the foveal depression forms [9,31,59]. During this phase we find a distinct peak of *Eph-A6* expression at the developing fovea (Figure 4 and Figure 5), with expression falling to lower levels either side of the fovea. Early in this period (Fd 105–120) the foveal avascular area is defined in the GCL, apparently by the migration of astrocytes being brought to a sudden halt on the edge of a ”no-go” zone that defines the fovea [9]. Our present findings suggest that high levels of *Eph-A6* expression in the GCL of the developing foveal region may have roles in driving capillaries deep into the GCL, and defining the no-go zone, by repelling ligand-bearing astrocytes. Further, these high levels of *Eph-A6* expression may explain the previously documented egress of astrocytes from the foveal region in the prenatal phase of monkey retinal development [9,60,61].

A distinctive feature of the perifoveal capillaries is their position in relation to the laminae of the neural retina. Throughout most of the primate retina, the innermost layer of vessels is found at the GCL-NFL interface. In the macula the innermost layer of capillaries forms at the GCL-IPL interface (Figure 4) [9,27,31], rather than more superficially at the GCL-NFL interface as in the rest of the retina. There are no known cues to explain the deep positioning of macular capillaries in the GCL. We propose that the *Eph-A6* gradient identified in the present study, which runs perpendicular to the retinal surface in the macular region, may explain the deep positioning of the macular capillaries. That is, we suggest that the high level of *Eph-A6* in the innermost cells of the GCL repels the migrating astrocytes into the deeper layers, where levels of *Eph-A6* mRNA are much lower. While the present data provide a sound basis for modeling the cellular interactions between GC and astrocytes that help to define the foveal avascular area, further in vitro studies are needed to validate and strengthen these hypotheses.

### A role in mapping macular ganglion cell projections?

The Eph–ephrin-A family has a key role in the mapping of GC projections onto the target visual nuclei [11,13], and it has been argued that the pattern of expression of the Eph–ephrin-A genes in the human retina may also contribute to the formation and maintenance of eye-specific projections to the laminae of the dorsal lateral geniculate nucleus [22]. Mapping of GC axons into broad territories in the visual targets takes place in macaques in the prenatal period [62]. While relatively little data are available concerning the postnatal period of GC axon-projection refinement in primates, it appears that this period of plasticity, during which preservation of synaptic territory of macular and foveal GC would be a priority, lasts until several months postnatal [32]. The sustained, high levels of expression of *Eph-A6* by macular GC in the postnatal period, described here, indicates that expression of *Eph-A6* may mark the projection from central retina during the postnatal phase of synaptic remodeling in the target visual nuclei. Such a role would predict reciprocal gradients of expression in the visual targets of the ligands *ephrin-A1* and *-A4*. We aim to undertake these experiments in the near future. Consistent with this, a role for *Eph-A6* in the axon projection to visual cortex has also been proposed, with *Eph-A6* expression localized to the site of visual cortex development in the macaque brain [63] before the arrival of thalamocortical projections [64].

### Conclusion

The present data implicates *Eph-A6* signaling in the development of retinal vessels prenatally, and suggests a role for *Eph-A6* in the preservation and maintenance of projections from macular and foveal GC, in the late prenatal and postnatal phase of development. In vitro analysis of interactions between *ephrin-A1* and –*A4* expressing astrocytes and retinal fragments with high versus low levels of *Eph-A6* expression are required to fully characterize the effects of *Eph-A6* signaling on astrocyte migration. Further investigation of the postnatal expression profiles of Eph–ephrin-As in the dorsal lateral geniculate nucleus, and superior colliculus, will clarify the roles these factors play in the postnatal refinement of retinal projections in primates.
